# Impact of sunlight exposure on the residual efficacy of biolarvicides *Bacillus thuringiensis israelensis* and *Bacillus sphaericus* against the main malaria vector, *Anopheles gambiae*

**DOI:** 10.1186/s12936-019-2687-0

**Published:** 2019-02-26

**Authors:** Barnabas Zogo, Bertin N’Cho Tchiekoi, Alphonsine A. Koffi, Amal Dahounto, Ludovic P. Ahoua Alou, Roch K. Dabiré, Lamine Baba-Moussa, Nicolas Moiroux, Cédric Pennetier

**Affiliations:** 1 Institut Pierre Richet (IPR), Bouaké, Côte d’Ivoire; 2 MIVEGEC, IRD, CNRS, Université de Montpellier, Montpellier, France; 3 Faculté des Sciences et Techniques/Université d’Abomey Calavi, Abomey-Calavi, Benin; 4 Institut de Recherche en Sciences de la Santé (IRSS), Bobo Dioulasso, Burkina Faso

**Keywords:** *Bacillus thuringiensis israelensis*, *Bacillus sphaericus*, Residual efficacy, Sunlight, Larval instars

## Abstract

**Background:**

Biotic and abiotic factors have been reported to affect the larvicidal efficacy of *Bacillus thuringiensis israelensis* (*Bti*) and *Bacillus sphaericus* (*Bs*), although the extent to which they are affected has been poorly documented. This paper studies the effect of sunlight exposure on the efficacy of a new larvicide formulation based on both *Bti* and *Bs*, herein after referred to as BTBSWAX, applied against two different larval stages.

**Methods:**

The emergence of inhibition exhibited by BTBSWAX at three different dosages (1 g/m^2^, 1.5 g/m^2^, and 2 g/m^2^) was monitored under semi-field conditions using a total of 32 containers comprising 16 that were covered and 16 that were uncovered. Two experiments were conducted using first- and second-instar larvae of *Anopheles gambiae*, respectively.

**Results:**

BTBSWAX at 2 g/m^2^ in covered containers exhibited high emergence inhibition (> 80%) when larvae were exposed from 1st instar on day-6 post-treatment, whereas the emergence inhibition was only 28% in uncovered containers. For larvae exposed from 1st instar on day-12 post-treatment, the emergence inhibition was moderate (70%) in covered containers but was low (< 20%) in uncovered containers. For larvae exposed from 2nd instar on day-10 post-treatment, the emergence inhibition was moderate (31%) in covered containers but was very low (< 10%) in uncovered containers. Moreover, the residual efficacy of BTBSWAX was markedly affected by environmental stresses, including sunlight exposure (Hazard ratio (HR) = 0.12, p < 0.001 and HR = 0.63, p = 0.033 for BTBSWAX at 2 g/m^2^ against 1st and 2nd instar larvae, respectively).

**Conclusion:**

These findings emphasize the impact of environmental variables (e.g., sunlight exposure) on the residual efficacy of *Bti* and *Bs* biolarvicides in the field. They hence highlight the need to take these factors into account for larvicide formulation development processes. Moreover, studies of the ecology of *Anopheles* larvae in targeted areas are also crucial for the integration of larval control strategies into malaria transmission plans devised by national malaria control programmes of endemic countries.

**Electronic supplementary material:**

The online version of this article (10.1186/s12936-019-2687-0) contains supplementary material, which is available to authorized users.

## Background

Vector control is a key component of the malaria control strategies that have led to the remarkable achievements made over the past decade [1]. However, the core vector control tools, namely insecticide-treated nets (ITNs) and indoor residual spraying (IRS) have clear limitations due to the increasing insecticide and behavioural resistance in vector populations [2–5]. According to the recent World Malaria Report, progress in malaria control has stalled in some areas since 2014 and additional tools are desperately needed if control is to result in elimination [6]. In Africa, the use of larval source management (LSM) as an additional tool for integrated vector management (IVM) has become increasingly relevant in recent years. LSM could be a valuable supplement to front-line tools, especially for the control of resistant vector populations. It has been an integral part of the vector control arsenal used worldwide to successfully eliminate malaria [7]. LSM strategies encompass habitat modification, habitat manipulation, biological control, and larviciding. Larviciding is a vector control intervention that consists of a regular application of chemical or biological insecticides in order to kill mosquito larvae.

*Bacillus thuringiensis* var *israelensis* (*Bti*) and *Bacillus sphaericus* (*Bs*) are the two potential biological insecticides that have been used for mosquito control since their discoveries during the mid-1970s. They are highly selective and are therefore environmentally safe to non-target organisms [8]. Moreover, to become resistant to *Bti*, an individual must develop resistance mechanisms to each of the four toxins, namely Cry4Aa, Cry4Ba, Cry11Aa, and Cyt1A [9]. The probability of development of resistance to *Bti* in the field is, therefore, very low, as opposed to virtually all of the single-target insecticides. To date, there have been no reports of resistance to *Bti* in mosquito populations despite it having been applied for decades in several countries [9, 10]. Laboratory studies have shown that Cyt1A proteins interact synergistically with Cry4A, Cry4B, and Cry11A and that they suppress cross-resistance to Cry toxins [11]. However, three studies have reported a reduced susceptibility to *Bti* in *Aedes*, *Culex,* and even *Anopheles* populations [12–14]. Unlike *Bti*, which requires clean water to be effective, *Bs* can provide good control of larvae in polluted habitats [15]. Therefore, there is a considerable advantage to use *Bs* as there is increasing evidence that *Anopheles gambiae* larvae are able to breed and develop in dirty and polluted habitats, especially in urban areas [15]. However, there have been several reports of high resistance to *Bs* in many areas [16–21], indicating that resistance management strategies are needed in operational programmes that use *Bs*. A mixture of *Bti* and *Bs* may represent a potentially effective approach and may prevent resistance to biolarvicides as insects are less likely to develop resistance to a mixture of toxins than to a single toxin [22]. The use of a mixture of *Bti* and *Bs* is also particularly useful for general mosquito abatement programmes, since the insecticidal activity of the two bacterial larvicides varies according to the mosquito species [23].

Several formulations of *Bti* and *Bs* such as water dispersible powder (WDP), wettable powder (WP), flowable concentrate (FC), emulsified concentrate (EC), dust, granules, and microencapsulated forms have been developed and tested under laboratory and field conditions [24–29]. Unfortunately, *Bti* and *Bs* quickly settle to the bottom after application, which reduces the quantity of larvicide present in the larval feeding zone [30]. Although the residual efficacy differs between formulations, biolarvicides—especially *Bti*—generally have a short residual efficacy in the field and hence their use requires frequent reapplication, thereby resulting in high operational costs [27, 31]. Some biotic and abiotic factors such as the mosquito species, the rate of ingestion, the density and age of the larvae, the temperature, the turbidity, and the organic matter content have been reported to affect the efficacy of biolarvicide formulations in the field [23, 32]. There has been a paucity, however, of trials that simultaneously assessed the effect of these factors on the efficacy of novel biolarvicide formulations. In this study, we assessed the effect of an environmental variable (e.g., sunlight exposure) on the residual efficacy of a new formulation mixing *Bti* and *Bs.*

## Methods

### Mosquito strain

The Kisumu strain of *An. gambiae* sensu stricto was used for this study. It is collected in Kenya and it is free of any detectable insecticide resistance mechanism [33]. This strain has been maintained for many years at controlled temperature (27 ± 2 °C) and relative humidity (70–80%) in the insectary of the Institut Pierre Richet (IPR) in Bouaké, central Côte d’Ivoire.

### Biolarvicide formulation

BTBSWAX (ISCA Technologies, Riverside, CA, USA) contains 83.3 g of *B. thuringiensis* Berliner 1915 subsp. *israelensis* (serovar H14, henceforth *Bti*) and 83.3 g of *B. sphaericus* (serotype H5a5b, strain 2362, henceforth *Bs*) per kilogramme of formulation. The wax has been manufactured with the same technology as SPLAT but it does not contain any pheromone lure nor any attractant. BTBSWAX floats on the water surface and hence does not settle to the bottom after application. Prior to application, the dollops were dried for 5 days in darkness at room temperature to prevent the development of an oily film on the water surface after treatment.

### Study design

The study was carried out between March 11th and April 4th, 2017 in an open sunlit area of IPR. Two experiments were conducted, each exposing either first- or second-instar larvae of *An. gambiae*. A total of 32 artificial cement containers (i.e., trays that were 30 cm deep × 50 cm long × 50 cm wide) that were spaced 50 cm from each other were used in the experiments. They were protected by a section of mosquito netting to prevent oviposition by wild female mosquitoes and the accumulation of debris. The study was designed according to the experimental set up of Djènontin et al. [24], with modifications to assess the effect of exposure to sunlight on the residual efficacy of the larvicide. The most important modification was the use of special stools (35 high × 90 cm long × 90 cm wide) draped with black plastic sheeting to cover 16 containers. Three dosages of fresh BTBSWAX (1 g/m^2^, 1.5 g/m^2^, and 2 g/m^2^) were evaluated in the uncovered containers (i.e., exposed to sunlight) and the covered containers (i.e., not exposed to sunlight). Overall, each experiment included the following treatments:

- Control (untreated) in four covered containers
- Control (untreated) in four uncovered containers
- BTBSWAX at 1 g/m^2^ in four covered containers
- BTBSWAX at 1 g/m^2^ in four uncovered containers
- BTBSWAX at 1.5 g/m^2^ in four covered containers
- BTBSWAX at 1.5 g/m^2^ in four uncovered containers
- BTBSWAX at 2 g/m^2^ in four covered containers
- BTBSWAX at 2 g/m^2^ in four uncovered containers

For each experiment, the various treatments were randomly assigned to the 32 containers (i.e., four per treatment) using the website Randomization.com.

### Experimental protocol

All of the containers were thoroughly cleaned, dried, and half-filled with 37.5 l of dechlorinated tap water prior to each experiment. The initial water level in the containers was maintained throughout the trial period. The appropriate amount of BTBSWAX (i.e., 250 mg, 375 mg, and 500 mg, corresponding to dosages of 1 g/m^2^, 1.5 g/m^2^, and 2 g/m^2^, respectively) was manually applied to the water surface. Freshly made dollops have a putty-like consistency. After weighing, the dollops were dried for 5 days in darkness at room temperature. In the first experiment, batches of 50 first-instar larvae were released in each container on day-0, 6, and 12 post-treatment, respectively. In the second experiment, second-instar larvae were released in each container on day-0, 10, and 21 post-treatment, respectively. Batches of 50 larvae were released on day-0 and 21 post-treatment and 30 larvae were released on day-10 post-treatment due to the limited number of larvae in the insectary. In each experiment, the frequency of the releases depended on the larval development period in the containers and the availability of larvae. Larvae were supplied with food (0.5 g of Tetramin© Baby per container) after they were released. After the treatment, all of the containers were checked twice on a daily basis (i.e., in the morning and in the afternoon) for the presence of pupae. All of the pupae were collected, counted, and placed into plastic cups with dechlorinated tap water. The plastic cups were covered with a section of netting until adult emergence. They were kept in the laboratory of IPR at room temperature until adult emergence. The number of adult mosquitoes collected from each plastic cup was recorded. The temperature and the pH of the water were monitored in each container on a daily basis (starting from 9 a.m.) using thermometers (Canac® 7080052, Quebec, Canada; accuracy 1 °C ± 0.2%) and reactive strips (Mel® 9800-6pk, Alon, Belgique; accuracy 1), respectively.

### Data analysis

The emergence rates of anopheles cohorts that had been exposed to the larvicide in the containers were compared to measure the residual efficacy of BTBSWAX. Statistical analyses were carried out with R software [34]. The variables were container-dependent. Two-tailed p values and a 5% significance level were used. The Emergence Inhibition Rates (% EIR) for each treatment were determined using the following formula:

$$\% {\text{EIR}} = \left( {\left( {{\text{C}} - {\text{T}}} \right)/{\text{C}}} \right) \times 100$$

where C is the emergence rate in the control and T is the emergence rate in the treated containers at the same point in time [35].

For each experiment, a dataset was created to be used for survival analysis (Additional files 1 and 2). This dataset had one line per larvae released with the final status of the larvae (1 = emerged; 0 = not emerged) and the time at which this status was recorded. The number of pupae counted on day *x* post release indicated the number of larvae with status “emerged” and time *x*. The number of larvae with status “not emerged” was obtained by subtracting, in each container and for each release, the total number of pupae counted to the number of larvae released. The data were right-censored such as larvae with status “not emerged” were associated with a time corresponding to the time of follow-up (i.e., for each release, the time at which the last pupae was observed). For each experiment (i.e. each larval instar tested), the risk of emergence was analysed using a Cox’s proportional hazards mixed-effect model with time after treatment, sunlight exposure (covered or uncovered), treatment (Control, 1 mg/m^2^, 1.5 mg/m^2^ or 2 mg/m^2^) and the interaction between sunlight exposure and treatment as fixed effects and containers random effect (random intercept). The “Coxme” function from the “coxme” package in ‘R’ was used [36, 37]. Kaplan–Meier plots of emergence were generated using the ‘survfit’ function in the library ‘survival’ (Additional file 3).

A linear mixed-effect model (function “lmer”, package “lme4”) was used to compare the water temperature in the covered versus the uncovered containers [38].

## Results

### Characteristics of the water

The water had a pH of 7 in all containers throughout both of the experimental periods (i.e. 1216 measures). During the first experiment, the measured temperatures ranged from 26 to 32 °C in the uncovered containers and the covered containers. During the second experiment, the measured temperatures ranged from 25 to 30 °C in the uncovered containers and from 25 to 29 °C in the covered containers. The linear mixed-effect model (“608 measures in covered containers *versus* 608 measures in uncovered containers”) showed that the water temperature was significantly higher in the uncovered containers than in the covered containers (df = 4636.01, 95% CI 0.40–0.51, p < 0.001) (Table 1).

**Table 1:** Summary of the temperatures measured during the experiments

Experiment	Instar	Containers	Minimum	Mean ± standard deviation	Maximum
1	2nd	Uncovered	26	28.6 ± 1.34	32
		Covered	26	27.9 ± 1.02	32
2	1st	Uncovered	25	27.9 ± 0.75	30
		Covered	25	27.7 ± 0.69	29

### Residual efficacy of BTBSWAX

**Fig. 1:**
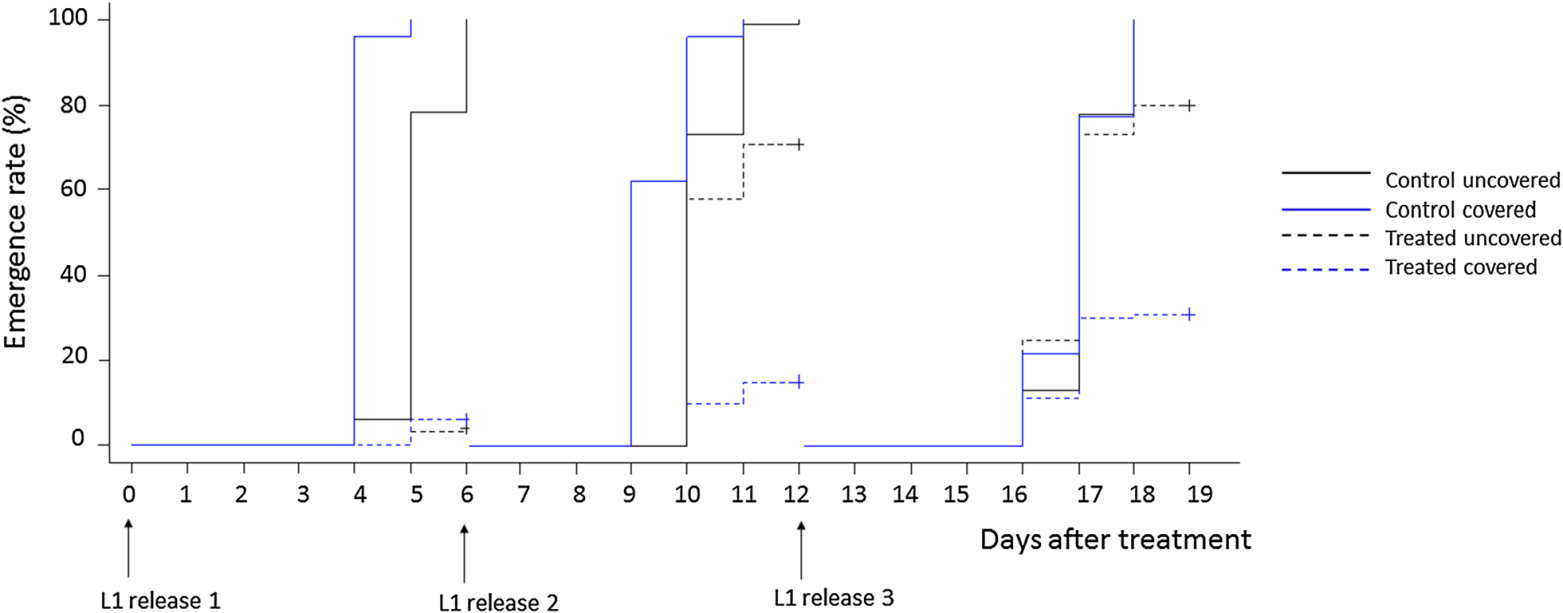
Emergence curves of first-instar larvae of *Anopheles gambiae* in treated (BTBSWAX at 2 g/m^2^) and control containers for the analysis of the effect of sunlight exposure. *L1* first-instar larvae

In the two experiments, the emergence rates of larvae in the negative control (untreated containers) were high, ranging from 98 to 100% and 83 to 100%, respectively (Tables 2, 3). In both of the experiments, BTBSWAX at 1.5 g/m^2^ and 2 g/m^2^ inhibited the emergence by more than 80% after exposure of larvae on day-0 post-treatment in covered and uncovered containers (Tables 2, 3). In covered containers, BTBSWAX at 2 g/m^2^ caused emergence inhibition of more than 80% after exposure of first-instar larvae released on day-6 post-treatment, whilst the emergence inhibition was only 28% in uncovered containers (Fig. 1). For first-instar larvae released on day-12 post-treatment, BTBSWAX resulted in a low level of emergence inhibition (EIR of 20%) in uncovered containers but still provided a high level of control (EIR of 70%) in covered containers (Fig. 1).

**Table 2:** Emergence and emergence inhibition rates after exposure of first-instar larvae of *Anopheles gambiae* to the treatments

N day post-treatment	Covered containers				Uncovered containers			
	Control	1 g/m^2^	1.5 g/m^2^	2 g/m^2^	Control	1 g/m^2^	1.5 g/m^2^	2 g/m^2^
0								
N	200	200	200	200	200	200	200	200
NE	200	17	10	14	200	118	33	10
ER (% [95% CI])	100 [98–100]	9 [5–13]	5 [3–7]	7 [4–10]	100 [98–100]	59 [52–66]	17 [12–22]	5 [2–8]
EIR (%)	–	92	95	93	–	42	84	95
6								
N	200	200	200	200	200	200	200	200
NE	200	21	40	32	200	186	110	145
ER (% [95% CI])	100 [98–100]	11 [7–15]	20 [15–25]	16 [12–20]	100 [98–100]	93 [89–97]	55 [48–62]	73 [66–80]
EIR (%)	–	90	80	84	–	7	45	28
12								
N	200	200	200	200	200	200	200	200
NE	200	88	92	60	200	195	182	161
ER (% [95% CI])	100 [98–100]	44 [37–51]	46 [39–53]	30 [24–36]	100 [98–100]	98 [94–100]	91 [86–96]	81 [74–88]
EIR (%)	–	56	54	70	-	3	9	20

*N* the number of larvae, *NE* the number of larvae emerged, *ER* the emergence rate, *EIR* the emergence inhibition rate

**Table 3:** Emergence and emergence inhibition rates after exposure of second-instar larvae of *Anopheles gambiae* to the treatments

N day post-treatment	Covered containers				Uncovered containers			
	Control	1 g/m^2^	1.5 g/m^2^	2 g/m^2^	Control	1 g/m^2^	1.5 g/m^2^	2 g/m^2^
0								
N	200	200	200	200	200	200	200	200
NE	191	23	20	18	171	40	25	1
ER (% [95% CI])	96 [92–100]	12 [8–16]	10 [7–13]	9 [6–12]	86 [80–92]	20 [15–25]	13 [9–17]	1 [0–2]
EIR (%)	–	88	90	91	–	77	85	100
10								
N	120	120	120	120	120	120	120	120
NE	108	76	56	74	106	98	100	110
ER (% [95% CI])	90 [83–97]	63 [54–72]	47 [38–56]	62 [53–71]	88 [81–95]	82 [74–90]	83 [76–90]	92 [85–99]
EIR (%)	–	30	48	31	–	8	6	0
21								
N	200	200	200	200	200	200	200	200
NE	189	183	177	180	200	195	186	196
ER (% [95% CI])	95 [91–99]	92 [88–96]	89 [85–93]	90 [86–94]	100 [98–100]	98 [96–100]	93 [91–95]	98 [95–100]
EIR (%)	–	3	6	5	–	3	8	3

*N* the number of larvae, *NE* the number of larvae emerged, *ER* the emergence rate, *EIR* the emergence inhibition rate

**Fig. 2:**
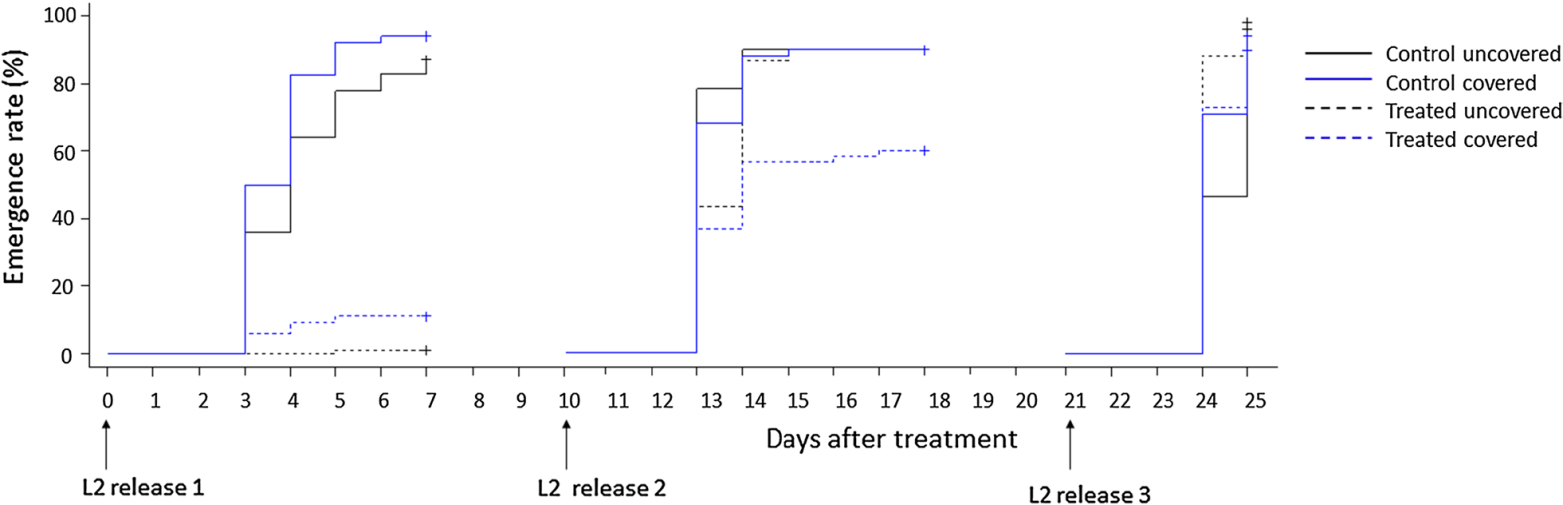
Emergence curves of second-instar larvae of *Anopheles gambiae* in treated (BTBSWAX at 2 g/m^2^) and control containers for the analysis of the effect of sunlight exposure. *L2* second-instar larvae

When second-instar larvae were exposed on day-10 post-treatment, the emergence inhibition was moderate (31%) in covered containers but was very low (< 10%) in uncovered containers. There were no significant differences in the emergence rates between treated and untreated containers after exposure of second-instar larvae on day-21 post-treatment (Fig. 2).

The Cox’s proportional hazards mixed-effect models (“12 control pools versus 12 test pools”) showed that sunlight exposure decreased significantly the emergence probability of larvae in containers treated with BTBSWAX at 1.5 g/m^2^ (Hazard ratio (HR) = 0.18, P < 0.001 and HR = 0.70, P = 0.020 against 1st and 2nd instar larvae, respectively) and 2 g/m^2^ (HR = 0.12, P < 0.001 and HR = 0.63, P = 0.033 against 1st and 2nd instar larvae, respectively) (Tables 4, 5) both with first-instar larvae and second-instar larvae (Figs. 1, 2).

**Table 4:** Cox’s proportional hazards model analyses of the emergence of larvae exposed from first instar

	Estimate (se)	Hazard ratio	P value
Treatments			
Dosage 1 g/m^2^	− 0.15 (0.31)	0.86	6.3e−01
Dosage 1.5 g/m^2^	− 0.97 (0.31)	0.38	1.6e−03**
Dosage 2 g/m^2^	− 1 (0.31)	0.37	1.1e−03**
Protection from sunlight exposure	0.54 (0.31)	1.72	7.9e−02
Days after treatment	0.02 (0)	1.02	5.9e−06***
Interactions between treatments and protection from exposure			
Dosage 1 g/m^2^ in covered containers	− 2.6 (0.44)	0.07	3.6e−09***
Dosage 1.5 g/m^2^ in covered containers	− 1.69 (0.44)	0.18	1.3e−04***
Dosage 2 g/m^2^ in covered containers	− 2.08 (0.45)	0.12	3.2e−06***

** P < 0.01, *** P < 0.001

**Table 5:** Cox’s proportional hazards model analyses of the emergence of larvae exposed from second instar

	Estimate (se)	Hazard Ratio	P value
Treatments			
Dosage 1 g/m^2^	− 0.39 (0.18)	0.68	0.031*
Dosage 1.5 g/m^2^	− 0.29 (0.18)	0.75	0.11
Dosage 2 g/m^2^	− 0.38 (0.18)	0.68	0.040*
Protection from sunlight exposure	0.25 (0.18)	1.28	0.17
Days after treatment	0.08 (0)	1.08	0.000***
Interactions between treatment and protection from exposure			
Dosage 1 g/m^2^ in covered containers	− 0.4 (0.26)	0.86	0.13
Dosage 1.5 g/m^2^ in covered containers	− 0.6 (0.26)	0.70	0.020*
Dosage 2 g/m^2^ in covered containers	− 0.55 (0.26)	0.63	0.033*

* P < 0.05, *** P < 0.001

It is noteworthy that the emergence of first instar larvae exposed 12 days after treatment was more inhibited (relative to the control) than the emergence of second instar larvae exposed 10 days after treatment (Tables 2, 3).

## Discussion

The residual efficacy of BTBSWAX at 2 g/m^2^ against larvae exposed from first and second instars was less than 10 days in uncovered containers. This result fits well with those from previous studies that reported short residual efficacies of *Bti* and *Bs* [24, 27]. *Bti* products are expected to provide a shorter residual efficacy than *Bs* products since the spores of *Bs* persist longer under field conditions and may recycle in the gut of mosquito larvae after they die [39]. However, the recycling capacity of *Bs* has been shown to depend on the formulation, the mosquito species, and environmental factors [40]. Compared to our results, a much longer effective period of a similar formulation (> 5 weeks after treatment) was observed against *Culex quinquefasciatus* larvae in a recent laboratory study [41]. Sedimentation of spores out of larval feeding areas had been reported to contribute to the short residual efficacy of biolarvicides against *Anopheles* larvae [24]. This is unlikely, however, since BTBSWAX formulation floats on the water surface after application. These results may be explained by the fact that *Culex* larvae are generally more susceptible to biolarvicide products than *Anopheles* larvae, although the susceptibility of mosquito species to *Bti* and *Bs* appears to vary between formulations [42].

Young larvae are known to be much more susceptible to *Bti* and *Bs* than late larvae [42]. A trend similar to these literature results was found in this study but this might also be explained by the longer exposure period of first-instar larvae compared to second-instar larvae as only the emergence rate was measured. The experimental design used in the present study did not allow to test statistically this hypothesis because (1) the semi-field conditions (cement plots) did not allow to discriminate alive and dead larvae impeding a release of larvae batches at a higher frequency (i.e. every 2 days) or (2) first and second instar larvae were not exposed to treatments in the same experiment at the same time.

Since several larval instars often prevail in natural breeding habitats, a difference in the susceptibility between larval instars should be taken into account in a potential control strategy using biolarvicides. By doing so, it would be possible to tune the frequency of retreatments and/or the dosages in order to kill late larvae and hence maintain a good efficacy of *Bti* and *Bs* in the field [43]. It should be noted that operational costs must be taken into account in such approaches as available funds are limited in all malaria control interventions. Moreover, a mixture of biolarvicide and surface film has also been tested against *Anopheles* larvae and pupae. Surface films killed mosquito larvae and pupae by reducing the surface tension of the water and, unlike biolarvicides, are more effective against late stages [44]. It was reported that the mixture exhibited greater efficacy than each tool on its own [44]. Nevertheless, further evaluations of this tool should be undertaken in various settings to provide more evidence of its efficacy and cost-effectiveness.

Environmental stresses, including sunlight exposure, significantly decreased the residual efficacy of BTBSWAX. The residual efficacy of BTBSWAX against larvae exposed from first instar was reduced by > 6 days in uncovered containers compared to that in covered containers. Solar gamma radiation and ultraviolet (UV) light have been shown to affect the spore viability of biolarvicides. Among the UV components, UV-A (320–400 nm) and UV-B (280–320 nm) have been identified as being responsible for the photodegradation and loss of activity of biolarvicides under field conditions [45]. Furthermore, a previous study has proven that solar radiation exposure decreases the efficacy of *Bs* by 30% [46]. Therefore, there is considerable interest in the development of technologies that provide protection of biolarvicides against environmental damage, especially for the control of *An. gambiae*, which mainly breed in sunlight-exposed habitats [47]. A number of adjuvants such as a fluorescent whitening agent, Congo red, folic acid, molasses, lignin, cellulose, alginate, shellac, and yeast have been shown to help achieve better protection from UV light [29]. However, these adjuvants appear to affect microbial growth and spore germination and they are also unstable under field conditions. Moreover, some ultraviolet light absorbents such as dyes and fluorescent agents are possible carcinogens [29]. More promising is a report that a new mixture of *Bti* and *Bs* formulation is effective for 4–6 months after application under semi-field conditions [48]. This result is very encouraging since longer residual efficacy would result in lower operational costs. Nonetheless, the effectiveness of biolarvicides does not only depend on their residual efficacy. The efficacy of biolarvicides is known to be affected by other factors, such as the dynamics of the breeding sites and their colonization [24]. As an example, biolarvicides are readily washed away by rain, and hence even a longer residual efficacy is unlikely to improve their efficacy during the rainy season [49].

This study has some limitations. The use of mosquito netting to cover all containers (in order to avoid oviposition by wild mosquitoes) may have interfered with sunlight irradiance in uncovered containers and then could have lessened the impact of sunlight exposure on the residual efficacy of BTBSWAX. Furthermore, the study design did not allow to measure precisely the temperature (temperature variation during the day) with only one measurement per day in the morning, when difference between different water bodies are likely to be the lowest during the day. Consequently, negative results about the temperature influence on treatment efficacies must be interpreted with caution. Another improvement for future experiments on *Bti* or *Bs* based products would be to measure precisely solar irradiance.

In order to devise a LSM strategic plan, *Anopheles* larval habitats need to be identified and characterized at a local scale. Breeding habitats are known to be very numerous, especially in rural areas of Africa, and their productivity and distribution vary in space and time. The hypothesis underlying LSM strategy is that “treating the most productive breeding sites in conjunction with LN universal coverage will impact malaria transmission”. To do so, identification and characterization of the most predominant and productive habitats is crucial. Knowledge of their dynamics will help with devising a targeted approach in order to get the best cost-effectiveness trade-off [50]. After this ecological study step, operational research on the effectiveness of long-lasting biolarvicides in different areas is critical for providing evidence of their impact on malaria transmission and for providing the most relevant strategy for their use.

## Conclusions

This study confirms that *Bti* and *Bs* are highly potent against *An. gambiae* larvae, although they have a short residual efficacy. Furthermore, environmental stresses, including sunlight exposure, significantly decreased the residual efficacy of BTBSWAX. Studies of the ecology of *Anopheles* larvae in targeted areas are also crucial for the integration of larval control strategies into malaria transmission plans devised by national malaria control programmes of endemic countries.

## Additional files

**Additional file 1.** Dataset of the first experiment using first instar larvae.

**Additional file 2.** Dataset of the second experiment using second instar larvae.

**Additional file 3.** Codes for the data analysis and figures.

## Abbreviations

*Bti*: *Bacillus thuringiensis israelensis*
*Bs*: *Bacillus sphaericus*
*An.*: *Anopheles*
ITNs: insecticide-treated nets
IRS: indoor residual spraying
LSM: larval source management
IVM: integrated vector management
WDP: water dispersible powder
WP: wettable powder
FC: flowable concentrate
EC: emulsified concentrate
UV: ultraviolet
IPR: Institut Pierre Richet
SPLAT: Specialized Pheromone and Lure Application Technology
EIR: emergence inhibition rates
N: number of larvae
NE: number of larvae emerged
ER: emergence rate

## References

[1] Bhatt S, Weiss DJ, Cameron E, Bisanzio D, Mappin B, Dalrymple U (2015). The effect of malaria control on *Plasmodium falciparum* in Africa between 2000 and 2015. Nature.

[2] Mwangangi JM, Mbogo CM, Orindi BO, Muturi EJ, Midega JT, Nzovu J (2013). Shifts in malaria vector species composition and transmission dynamics along the Kenyan coast over the past 20 years. Malar J..

[3] Moiroux N, Gomez MB, Pennetier C, Elanga E, Djènontin A, Chandre F (2012). Changes in *Anopheles funestus* biting behavior following universal coverage of long-lasting insecticidal nets in Benin. J Infect Dis.

[4] WHO (2014). Control of residual malaria parasite transmission.

[5] Ranson H, Lissenden N (2016). Insecticide resistance in African Anopheles mosquitoes: a worsening situation that needs urgent action to maintain malaria control. Trends Parasitol..

[6] WHO (2017). World malaria report.

[7] WHO (2013). Larval source management—a supplementary measure for malaria vector control An operational manual.

[8] Ketseoglou I, Koekemoer LL, Coetzee M, Bouwer G (2011). The larvicidal efficacy of *Bacillus thuringiensis* subsp. *israelensis* against five African Anopheles (Diptera: Culicidae) species. Afr Entomol..

[9] Vasquez MI, Violaris M, Hadjivassilis A, Wirth MC (2009). Susceptibility of *Culex pipiens* (Diptera: Culicidae) field populations in Cyprus to conventional organic insecticides, *Bacillus thuringiensis* subsp. *israelensis*, and methoprene. J Med Entomol..

[10] Becker N, Ludwig M (1993). Investigations on possible resistance in *Aedes vexans* field populations after a 10-year application of *Bacillus thuringiensis israelensis*. J Am Mosq Control Assoc..

[11] Georghiou GP, Wirth MC (1997). Influence of exposure to single versus multiple toxins of *Bacillus thuringiensis* subsp. *israelensis* on development of resistance in the mosquito *Culex quinquefasciatus* (Diptera: Culicidae). Appl Environ Microbiol..

[12] Hongyu Z, Changju Y, Jingye H, Lin L (2004). Susceptibility of field populations of *Anopheles sinensis* (Diptera: Culicidae) to *Bacillus thuringiensis* subsp. *israelensis*. Biocontrol Sci Technol..

[13] Paul A, Harrington LC, Zhang L, Scott JG (2005). Insecticide resistance in *Culex pipiens* from New York. J Am Mosq Control Assoc..

[14] Boyer S, Paris M, Jego S, Lempérière G, Ravanel P (2012). Influence of insecticide *Bacillus thuringiensis* subsp. *israelensis* treatments on resistance and enzyme activities in *Aedes rusticus* larvae (Diptera: Culicidae). Biol Control..

[15] Walker K, Lynch M (2007). Contribution of Anopheles larvae control to malaria suppression in tropical Africa: review of achievements and potential. Med Vet Entomol.

[16] Chevillon C, Bernard C, Marquine M, Pasteur N (2001). Resistance to *Bacillus sphaericus* in *Culex pipiens* (Diptera: Culicidae): interaction between recessive mutants and evolution in southern France. J Med Entomol.

[17] Zahiri NS, Su T, Mulla MS (2002). Strategies for the management of resistance in mosquitoes to the microbial control agent *Bacillus sphaericus*. J Med Entomol.

[18] Nielsen-Leroux C, Charles JF, Thiéry I, Georghiou GP (1995). Resistance in a laboratory population of *Culex quinquefasciatus* (Diptera: Culicidae) to *Bacillus sphaericus* binary toxin is due to a change in the receptor on midgut brush-border membranes. Eur J Biochem.

[19] Poopathi S, Mani TR, Rao DR, Baskaran G, Kabilan L (1999). Cross-resistance to *Bacillus sphaericus* strains in *Culex quinquefasciatus* resistant to *B sphaericus* 1593M. Southeast Asian J Trop Med Public Health..

[20] Rao DR, Mani TR, Rajendran R, Joseph AS, Gajanana A, Reuben R (1995). Development of a high level of resistance to *Bacillus sphaericus* in a field population of *Culex quinquefasciatus* from Kochi, India. J Am Mosq Control Assoc..

[21] Adak T, Mittal PK, Raghavendra K, Subbarao SK, Ansari MA, Sharma VP (1995). Resistance to *Bacillus sphaericus* in *Culex quinquefasciatus* Say 1823. Curr Sci.

[22] Pennetier C, Costantini C, Corbel V, Licciardi S, Dabiré RK, Lapied B (2008). Mixture for controlling insecticide-resistant malaria vectors. Emerg Infect Dis.

[23] Lacey LA (2007). *Bacillus thuringiensis* serovariety *israelensis* and *Bacillus sphaericus* for mosquito control. J Am Mosq Control Assoc..

[24] Djènontin A, Pennetier C, Zogo B, Soukou KB, Ole-Sangba M, Akogbéto M (2014). Field efficacy of vectobac GR as a mosquito larvicide for the control of anopheline and culicine mosquitoes in natural habitats in Benin, West Africa. PLoS One..

[25] Fillinger U, Bv I, Becker N (2003). Efficacy and efficiency of new *Bacillus thuringiensis* var. *israelensis* and *Bacillus sphaericus* formulations against Afrotropical anophelines in Western Kenya. Trop Med Int Health..

[26] Davidson EW, Sweeney AW, Cooper R (1981). Comparative field trials of *Bacillus sphaericus* strain 1593 and *B. thuringiensis* var. *israelensis* commercial powder formulations. J Econ Entomol..

[27] Skovmand O, Sanogo E (1999). Experimental formulations of *Bacillus sphaericus* and *B. thuringiensis israelensis* against *Culex quinquefasciatus* and *Anopheles gambiae* (Diptera: Culicidae) in Burkina Faso. J Med Entomol..

[28] Gunasekaran K, Prabakaran G, Balaraman K (2002). Efficacy of a floating sustained release formulation of *Bacillus thuringiensis* ssp. *israelensis* in controlling *Culex quinquefasciatus* larvae in polluted water habitats. Acta Trop..

[29] Zhang L, Zhang X, Zhang Y, Wu S, Gelbič I, Xu L (2016). A new formulation of *Bacillus thuringiensis*: UV protection and sustained release mosquito larvae studies. Sci Rep..

[30] Mittal PK (2003). Biolarvicides in vector control: challenges and prospects. J Vect Borne Dis..

[31] Fillinger U, Ndenga B, Githeko A, Lindsay SW (2009). Integrated malaria vector control with microbial larvicides and insecticide-treated nets in western Kenya: a controlled trial. Bull World Health Organ.

[32] Becker N, Zgomba IM, Ludwig M, Petric D, Rettich F (1992). Factors influencing the activity of *Bacillus thuringiensis* var. *israelensis* treatments. J Am Mosq Control Assoc..

[33] Shute GT (1956). A method of maintaining colonies of East African strains of *Anopheles gambiae*. Ann Trop Med Parasitol.

[34] R Core Team. R: a language and environment for statistical computing. R foundation for statistical computing. Vienna, Austria. https://www.R-project.org/. 2018.

[35] WHO Communicable Disease Control Prevention and Eradication (2005). Guidelines for laboratory and field testing of mosquito larvicides.

[36] Therneau T. Coxme. Mixed effects cox models. R package version 2.2-10; 2018. https://CRAN.R-project.org/package=coxme. Accessed 18 June 2018.

[37] Therneau T. A package for survival analysis in S, version 2.38; 2015. https://CRAN.R-project.org/package=survival. Accessed 18 June 2018.

[38] Bates D, Maechler M, Bolker B, Walker S (2015). Fitting linear mixed-effects models using lme4. J Stat Softw.

[39] Becker N, Zgomba M, Petric D, Beck M, Ludwig M (1995). Role of larval cadavers in recycling processes of *Bacillus sphaericus*. J Am Mosq Control Assoc..

[40] Mwangangi JM, Kahindi SC, Kibe LW, Nzovu JG, Luethy P, Githure JI (2011). Wide-scale application of Bti/Bs biolarvicide in different aquatic habitat types in urban and peri-urban Malindi, Kenya. Parasitol Res..

[41] Schorkopf DLP, Spanoudis CG, Mboera LEG, Mafra-Neto A, Ignell R, Dekker T (2016). Combining attractants and larvicides in biodegradable matrices for sustainable mosquito vector control. PLoS Negl Trop Dis..

[42] Charles JF, Nielsen-LeRoux C (2000). Mosquitocidal bacterial toxins: diversity, mode of action and resistance phenomena. Mem Inst Oswaldo Cruz.

[43] Mulla MS, Darwazeh HA, Zgomba M (1995). Effect of some environmental factors on the efficacy of *Bacillus sphaericus* 2362 and *Bacillus thuringiensis* (H-14) against mosquitoes. Bull Soc Vector Ecol..

[44] Bukhari T, Takken W, Koenraadt CJM (2013). Biological tools for control of larval stages of malaria vectors—a review. Biocontrol Sci Technol..

[45] Manasherob R, Ben-Dov E, Xiaoqiang W, Boussiba S, Zaritsky A (2002). Protection from UV-B damage of mosquito larvicidal toxins from *Bacillus thuringiensis* subsp. *israelensis* expressed in Anabaena PCC 7120. Curr Microbiol..

[46] Rojas JE, Mazzarri M, Sojo M, García-A GY (2001). Effectiveness of *Bacillus sphaericus* strain 2362 on larvae of *Anopheles nuñeztovari*. Invest Clin.

[47] Minakawa N, Mutero CM, Githure JI, Beier JC, Yan G (1999). Spatial distribution and habitat characterization of anopheline mosquito larvae in western Kenya. Am J Trop Med Hyg.

[48] Afrane YA, Mweresa NG, Wanjala CL, Gilbreath TM, Zhou G, Lee M-C (2016). Evaluation of long-lasting microbial larvicide for malaria vector control in Kenya. Malar J..

[49] Fillinger U, Lindsay SW (2006). Suppression of exposure to malaria vectors by an order of magnitude using microbial larvicides in rural Kenya. Trop Med Int Health..

[50] Gu W, Novak RJ (2005). Habitat-based modeling of impacts of mosquito larval interventions on entomological inoculation rates, incidence, and prevalence of malaria. Am J Trop Med Hyg.

